# Identification of LINC00665-miR-let-7b-*CCNA2* competing endogenous RNA network associated with prognosis of lung adenocarcinoma

**DOI:** 10.1038/s41598-020-80662-x

**Published:** 2021-02-24

**Authors:** Yusheng Huang, Limei Zhong, Kechao Nie, Lijuan Li, Shaohua Song, Fengbin Liu, Peiwu Li, Donglin Cao, Yufeng Liu

**Affiliations:** 1 Department of Gastroenterology, The First Affiliated Hospital of Guangzhou University of Chinese Medicine, No. 12 Airport Road, Baiyun District, Guangzhou, 510407 China; 2 Department of Laboratory Medicine, Guangdong Second Provincial General Hospital, Guangzhou, 510317 China; 3 Lingnan Medical Research Center, Guangzhou University of Chinese Medicine, Guangzhou, 510407 China

**Keywords:** Non-small-cell lung cancer, Lung cancer, Computational biology and bioinformatics

## Abstract

Prognosis of patients with lung cancer remains extremely poor; thus, we sought to unearth novel competing endogenous RNA (ceRNA) networks associated with the prognosis of lung adenocarcinoma (LUAD). Aberrant mRNAs were identified from the intersection of three Gene Expression Omnibus (GEO) datasets. A protein–protein interaction (PPI) network was constructed, and miRNAs and long noncoding RNAs (lncRNAs) upstream of mRNAs were predicted. In the present study, 402 upregulated and 638 downregulated genes in lung cancer tissues were identified. Functional analysis showed significant enrichment of cancer pathways. In these top hub genes, 10 upregulated and 7 downregulated genes had substantial prognostic values in LUAD. Thirty-seven miRNAs were predicted to target 17 key genes, and only five miRNAs exhibited prognostic correlation. Through stepwise reverse prediction and validation from miRNA to lncRNA, four key lncRNAs were identified using expression and survival analysis. Ultimately, the co-expression analysis identified LINC00665-miR-let-7b-*CCNA2* as the key ceRNA network associated with the prognosis of LUAD. We successfully constructed a novel ceRNA network wherein each component was significantly associated with the prognosis of LUAD. Hence, we propose that this network may provide key biomarkers or potential therapeutic targets for LUAD prognosis.

## Introduction

Lung cancer remains the leading cause of cancer-related death worldwide, accounting for 19.4% of overall cancer mortality^1^. Notably, most lung cancer patients have a poor prognosis^2^. Primary lung cancer is generally divided into small cell lung cancer (SCLC) and non-small cell lung cancer (NSCLC). NSCLC accounts for about 80% of all primary lung cancers, of which adenocarcinoma, squamous cell carcinoma, and large cell carcinoma are the main histological subset^3^. Despite improvements and advancements in early diagnostic and treatment methods, the 5-year survival rate for primary lung cancer is still as low as 11–16%^4,5^. With a deeper understanding of the molecular biological mechanisms of lung cancer, targeted therapy has made great progress.

Whole-genome analysis of gene expression shows that exons account for less than 3% of the human genome, and an increase in the number of noncoding RNA (ncRNA) genes in introns reportedly regulates gene expression^6^. ncRNAs include three types: long ncRNAs (lncRNAs), mid-size ncRNAs, and short ncRNAs. Among short ncRNAs, miRNAs target gene regulation and can affect lung cancer treatment^7^. Salmena et al. proposed the competing endogenous RNA (ceRNA) hypothesis whereby mRNAs, miRNAs, and lncRNAs could cross-talk, forming a regulatory network^8^. Through miRNA response elements, lncRNAs could be sequestering RNA-binding proteins and microRNAs leading to changes in the miRNA-regulated mRNA levels. Increasing evidence indicates that this ceRNA network plays a pivotal role in a variety of human cancers, such as breast cancer^9^, gastric cancer^10^, liver cancer^11^, and pancreatic cancer^12^.

Generally, lncRNAs consist of more than 200 nucleotides with limited or no protein-coding capacity. To date, more than 3000 lncRNAs have been identified. Abnormal expression of various lncRNAs is related to carcinogenesis. In particular, it can lead to the occurrence, development, and metastasis of multiple cancers, such as breast cancer, hepatocellular carcinoma, and cardiac adenocarcinoma^13–15^. In physiological conditions, lncRNAs participate in multiple biological processes, including splicing, transcription, epigenetic gene expression, and chromatin modification^16^.

Research has shown that the expression of HOTAIR, MALAT1, HOTTIP, ANRIL, and ZXF2 were upregulated in lung cancer tissues, which is related to increased tumor lymph node metastasis rate, advanced lymph node metastasis, and decreased overall survival^17–21^. Considering their function in the development of lung cancer, they could become promising biomarkers for the diagnosis or prognosis of lung cancer^22^. However, the current understanding of the key lncRNA–miRNA–mRNA ceRNA networks that are significantly related to the prognosis of lung cancer remains unclear.

In our work, we screened mRNAs that were differentially expressed (DE-mRNAs) in lung cancer tissues compared to healthy tissues by mining three Gene Expression Omnibus (GEO) datasets (GSE18842, GSE19188, and GSE33532). The common aberrantly expressed mRNAs were listed, and their functional enrichment analysis was carried out. Subsequently, we conducted protein–protein interaction (PPI) analysis using the STRING database and identified several up- and downregulated hub genes. Then, miRTarBase was used to predict the upstream miRNAs. Taking miRNA expression and prognostic values into consideration, candidate miRNAs were further selected to predict the potential upstream lncRNAs. Furthermore, the prognostic value of these potential lncRNAs was assessed, and the co-expression analysis between mRNAs, miRNAs, and lncRNAs was evaluated using the ceRNA hypothesis. Finally, we successfully established a novel ceRNA regulatory network, which was significantly associated with the prognosis of patients with LUAD. Our research sheds light on the onset of LUAD and identifies potential diagnostic biomarkers or therapeutic targets.

## Results

### Identification of significant differentially expressed genes (DEGs) in LUAD

**Figure 1:**
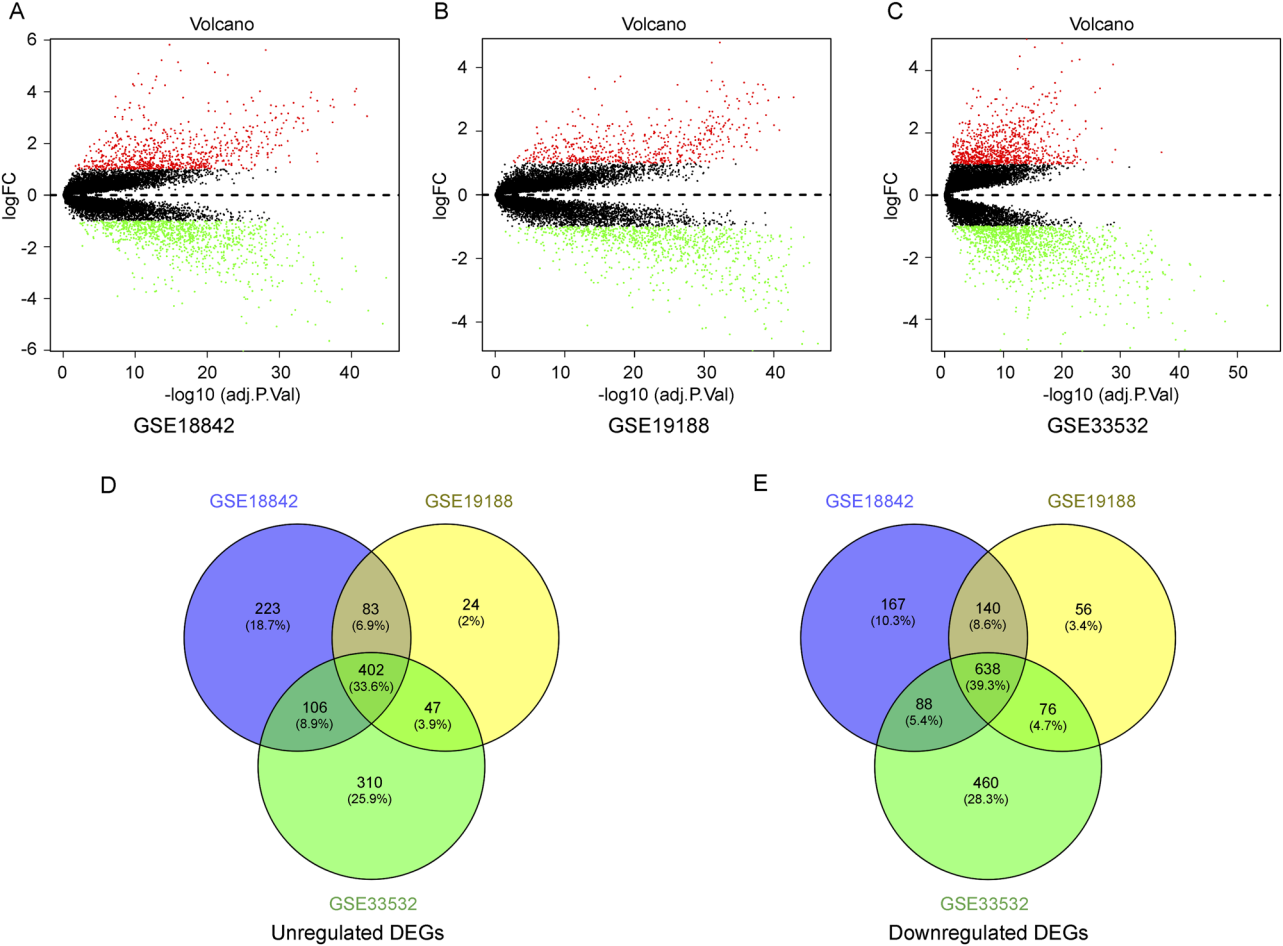
Identification of differentially expressed mRNAs between lung adenocarcinoma (LUAD) and normal samples in three Gene Expression Omnibus (GEO) datasets. (**A**–**C**) Volcano plots of DE-mRNAs in GSE18842, GSE19188, and GSE33532. Horizontal axis represents the − log_10_ (adjusted p-value) and vertical axis represents the log_2_ (fold change) of gene expression. Green dots and red dots represent significantly downregulated and upregulated genes, respectively. Black dots represent genes with no significant difference. (**D**, **E**) The intersection of upregulated and downregulated genes in three datasets, respectively. DEG; DE-mRNA: Differentially expressed mRNA.

Searching for gene expression microarrays with regard to LUAD from the GEO database, three datasets (GSE18842, GSE19188, and GSE33532) were selected, and information of three GEO data sets were shown in Table 1. Next, DEGs analysis was performed using GEO2R (|log_2_FC|> 1 and adj. p-value < 0.05), and significant DEGs in each dataset were identified (Fig. 1A–C). In the GSE18842 dataset, a total of 815 upregulated and 1034 downregulated mRNAs were screened. For the GSE19188 dataset, there were 557 upregulated and 911 downregulated genes in lung cancer tissues compared to those in normal control samples. Finally, in the GSE33532 dataset, 866 upregulated and 1263 downregulated genes were identified. After the upregulated or downregulated mRNAs from each dataset were intersected, we identified a total of 402 upregulated and 638 downregulated DE-mRNAs shared among the three datasets and selected them for subsequent analyses (Fig. 1D, E). The detailed DE-mRNAs are listed in Supplementary Excel 1–3.

**Table 1:** The gene expression datasets characteristics.

Dataset	Platform	Number of samples	Number of tumor/adjacent	Region
GSE18842	Affymetrix Human Genome U133 Plus 2.0 Array	91	46/45	Spain
GSE19188	Affymetrix Human Genome U133 Plus 2.0 Array	156	91/65	Netherlands
GSE33532	Affymetrix Human Genome U133 Plus 2.0 Array	100	80/20	Germany

### Functional analysis of the DE-mRNAs

**Figure 2:**
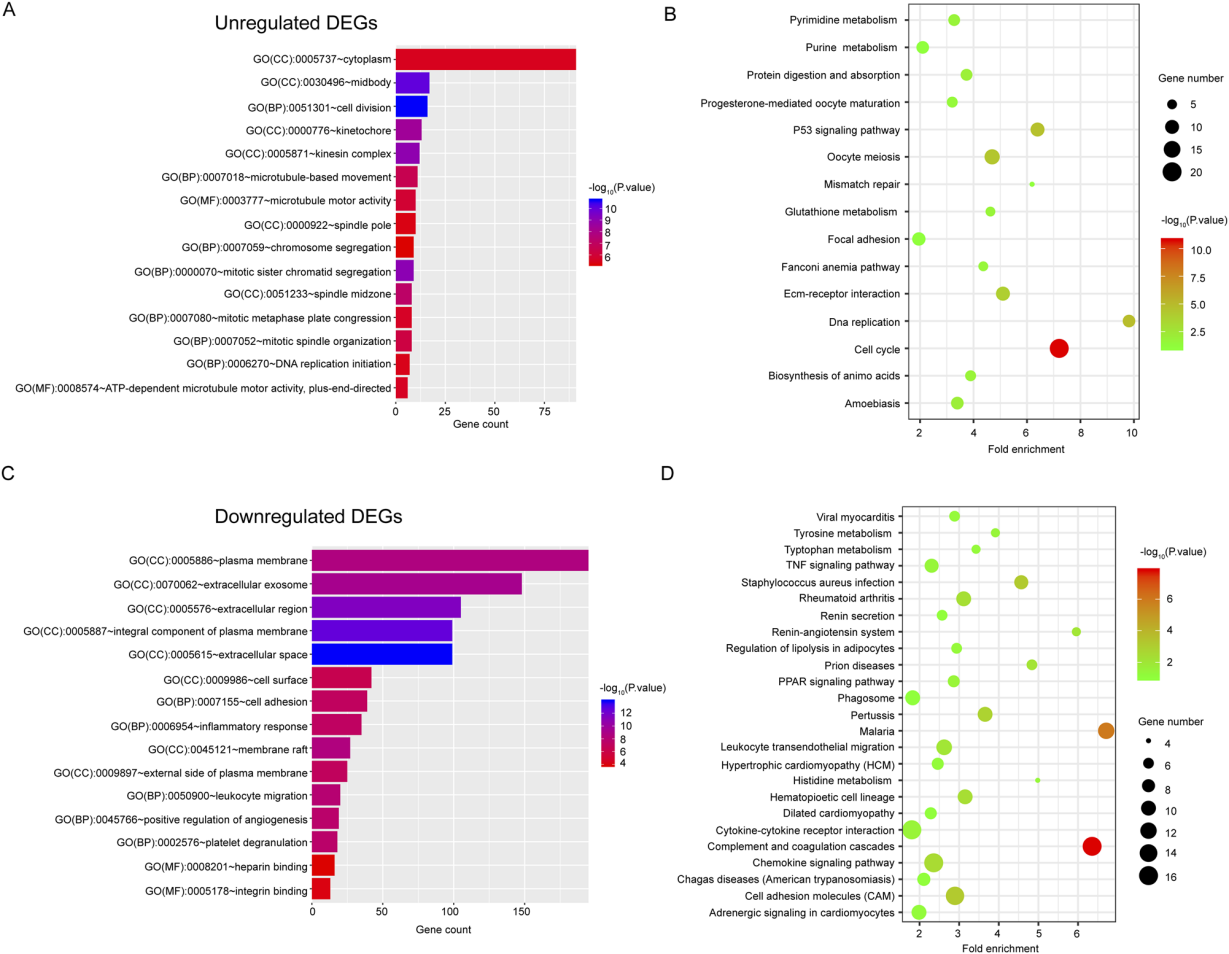
Functional and protein–protein interaction (PPI) networks analysis for the intersected differentially expressed mRNAs. (**A**, **C**) Enriched GO pathways of the significantly upregulated and downregulated genes, respectively. (**B**, **D**) Enriched KEGG pathways of the significantly upregulated and downregulated genes, respectively. *BP* biological process; *CC* cellular component; *MF* molecular function; *GO* Gene Ontology; *KEGG* Kyoto Encyclopaedia of Genes and Genome.

To predict the biological functions of the identified DE-mRNAs, we performed Gene Ontology (GO) enrichment and Kyoto Encyclopaedia of Genes and Genomes (KEGG) pathway analysis. Predicting the biological function of identified DE-mRNAs, we performed GO term enrichment and KEGG pathway analysis. Analysis of biological processes was mainly performed in the GO annotation. For upregulated DE-mRNAs, our results indicated that these genes were notably enriched in processes associated with cell division, DNA replication, and cytoskeletal movements (Fig. 2A).Additionally, cell cycle and malignancy-related pathways, such as the p53 signaling pathway and amino acid metabolism, were observed in the KEGG pathway analysis (Fig. 2B). Notably, positive regulation of angiogenesis was observed, and the biological process analysis also indicated several cancer-related GO terms, such as cell adhesion and extracellular region/space (Fig. 2C). Furthermore, the KEGG pathway analysis also found that some downregulated genes were significantly enriched in well-known cancer-associated pathways, including cell adhesion molecules, TNF, PPAR, and chemokine signaling pathways (Fig. 2D). Altogether, these results indicated that both up- and downregulated DE-mRNAs were closely related to lung cancer.

### Construction of the PPI network and identification of hub genes

STRING-based database analysis, Supplementary Figure 1a, c show significantly different PPI networks related to up- and downregulated DEGs, respectively. Based on the degree of each node, we identified 20 hub genes among these significant differences. For better visualization, we used the Cytoscape software to reconstruct the top 20 interacting genes of up- (Supplementary Figure 1B) and downregulated (Supplementary Figure 1D) hub genes. The top 20 hub genes that were upregulated and the top 20 hub genes that were downregulated were selected for subsequent analyses.

### Validation of gene expression of hub genes and survival analysis

**Figure 3:**
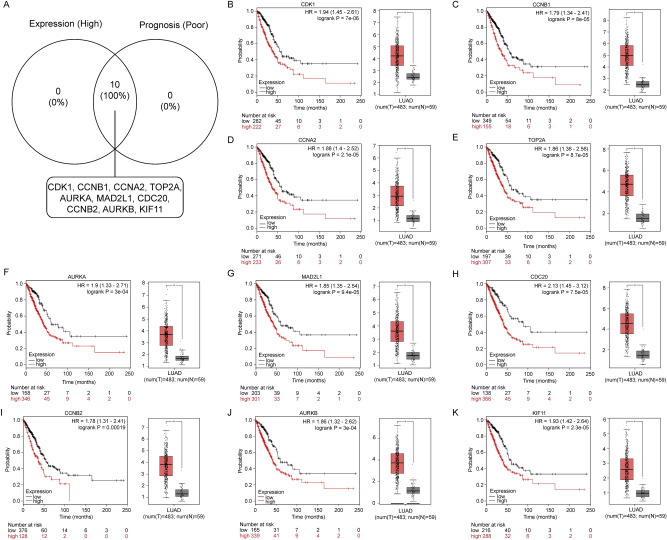
Screening the key upregulated genes in LUAD. (**A**) Identification of the key genes with high expression and poor prognosis values among significantly upregulated hub genes. (**B**–**K**) Representative expression and prognostic value of hub genes validated with Gene Expression Profiling Interactive Analysis (GEPIA) and Kaplan–Meier plotter databases, respectively.

**Figure 4:**
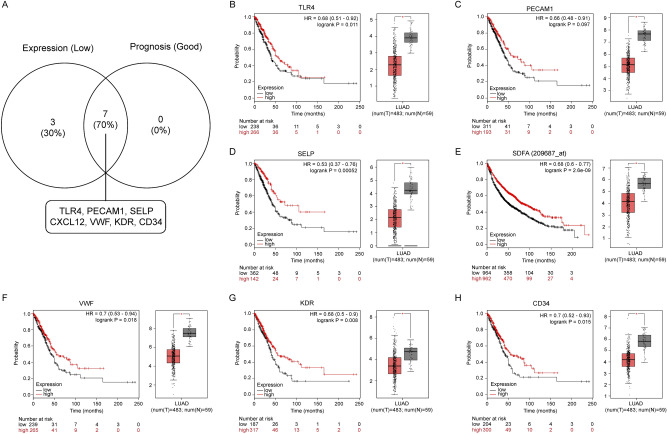
Screening the key downregulated genes in LUAD. (**A**) Identification of key genes with low expression and good prognosis values in significantly downregulated hub genes. (**B**–**H**) Representative expression and prognostic value of hub genes validated with GEPIA and Kaplan–Meier plotter databases, respectively.

Aiming to determine the expression of key genes in lung cancer, we used gene expression profiling analysis (GEPIA) and the Kaplan–Meier plotter database to analyze the expression and prognostic value of the top 10 up- and downregulated hub genes, respectively. Combining expression patterns and survival analysis, we found that 10 upregulated hub genes (CDK1, CCNB1, CCNA2, TOP2A, AURKA, MAD2L1, CDC20, CCNB2, AURKB, and KIF11) were not only significantly upregulated in lung cancer but also significantly correlated with poor prognosis of lung cancer patients (p < 0.05; Fig. 3A–K). Conversely, seven of the downregulated hub genes (TLR4, PECAM1, SELP, CXCL12, VWF, KDR, and CD34) showed both low expression and good prognosis in lung cancer patients (p < 0.05; Fig. 4A–H). These 17 key genes, which met the criteria of converse expression pattern and survival prognosis, were selected for the next analyses.

### Prediction and validation of key miRNAs upstream of critical genes

**Figure 5:**
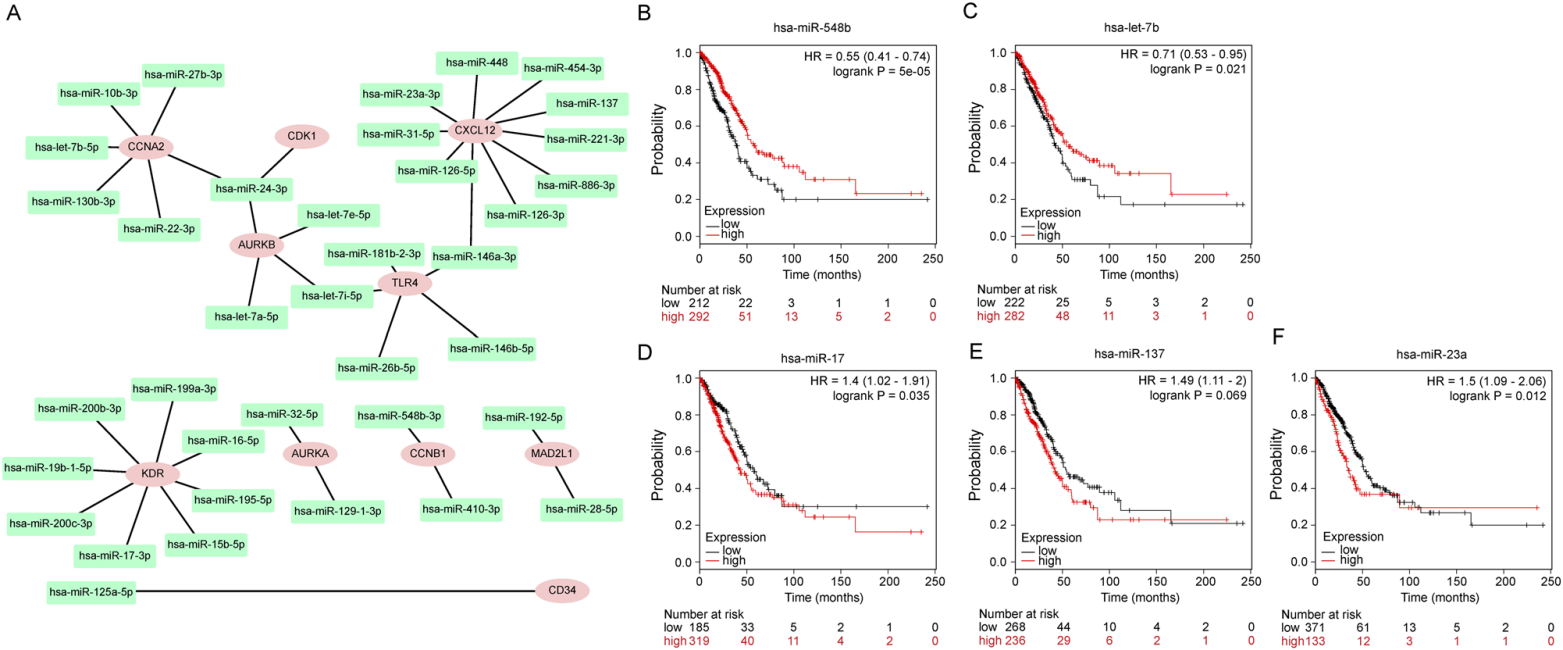
Construction of miRNA-gene network using Cytoscape software. (**A**) The rectangle in the network represents miRNA. The ellipse in the network represents hub genes. (**B**–**F**) Representative prognostic value of key miRNAs validated using Kaplan–Meier plotter databases.

In order to identify key miRNAs that regulate the pivotal hub genes, we predicted their upstream miRNAs by using the miRTarBase database, which is the experimentally validated miRNA-target interaction. Finally, we identified 37 miRNAs that may regulate the expression of key pivot genes, as shown in Fig. 5A. We downloaded the miRNA expression profile and survival information of lung adenocarcinoma patients from TCGA project. Then, we selected 36 out of 37 miRNAs obtained from TCGA project to construct a risk signature using multivariate Cox regression analysis. The risk score of each patient was based on a linear combination of miRNA expression level (X) multiplied by the regression coefficient (α) from the multivariate Cox regression analysis. The formula is as follows: risk score = X1α1 + X2α2 + X3α3 + ⋯ + Xnαn. Twelve miRNAs comprise the risk signature. The results showed that high-risk patients had a shorter survival time than patients with lung adenocarcinoma, while the risk signature was not a good prediction model for prognosis of lung adenocarcinoma patients (Supplementary Figure 2a, b). According to the negative regulatory relationship between miRNA and its target genes, we further evaluated the prognostic value of miRNA for overall survival in patients with LUAD using the Kaplan–Meier plotter database. Survival analysis showed that high expression of two downregulated miRNAs, miR-548b and miR-let-7b, functioned as better prognostic biomarkers in patients with LUAD (Fig. 5B, C). By contrast, three upregulated miRNAs, miR-17, miR-137, and miR-23a, displayed negative prognostic function in these patients (Fig. 5D–F). These miRNAs were identified as key regulators and were analyzed further.

### Prediction and validation of upstream key lncRNAs

**Figure 6:**
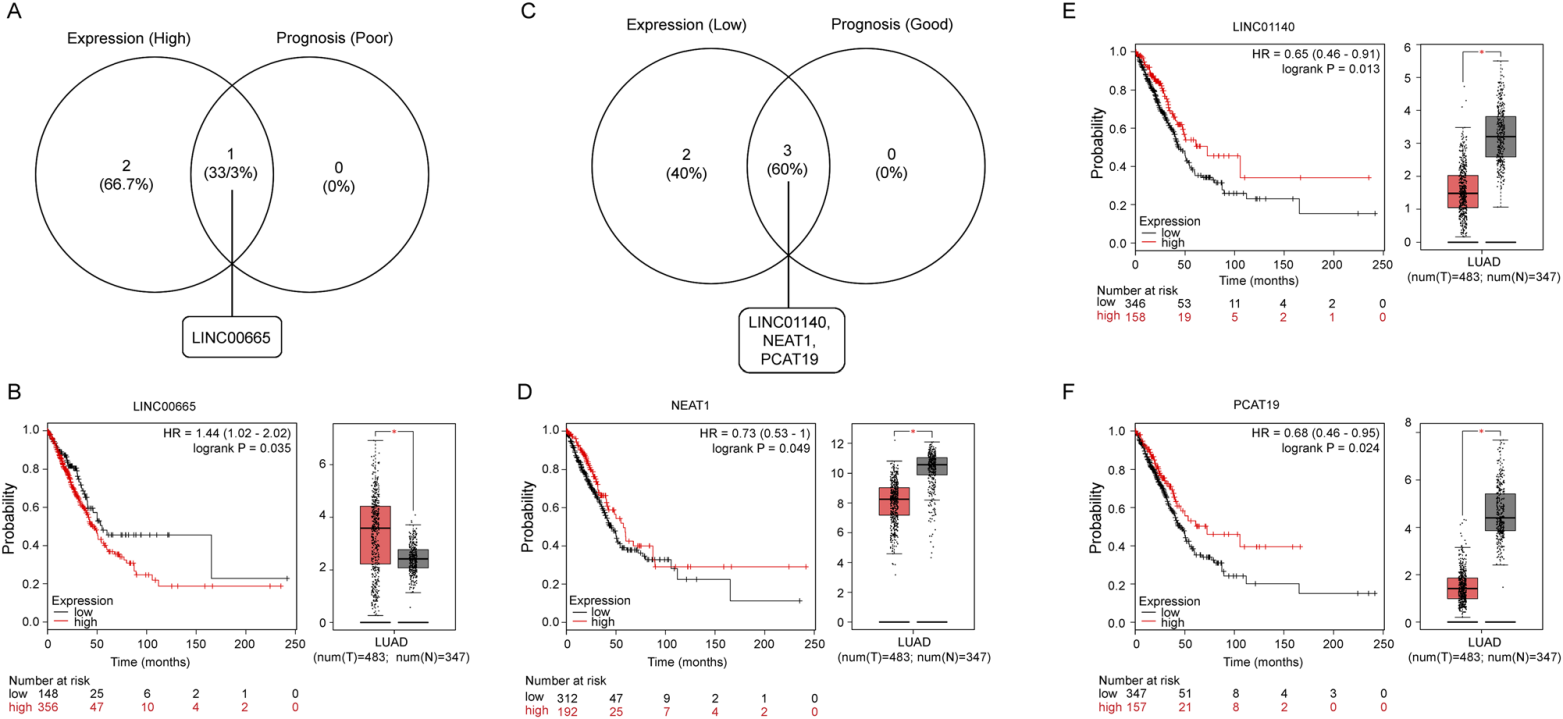
Screening the key lncRNAs in LUAD. (**A**, **B**) Identification of the key long noncoding RNAs (lncRNAs) with high expression and poor prognosis values among the predicted candidate lncRNAs by combining expression and prognosis analyses using GEPIA and Kaplan–Meier-plotter databases, respectively. (**C**–**F**) Identification of key lncRNAs with low expression and better prognosis values among predicted candidate lncRNAs by combining expression and prognosis analyses using GEPIA and Kaplan–Meier-plotter databases, respectively.

lncRNAs can regulate gene expression by influencing the transcription, mRNA turnover, and translation by sequestering RNA binding proteins and microRNAs^23^. We further used the online database, miRNet, to predict the lncRNAs that may bind to 5 key miRNAs (miR-548b, miR-let-7b, miR-17, miR-137, and miR-23a). A total of 198 lncRNAs were discovered in the database for the two upregulated miRNAs, while 426 lncRNAs were found to be able to potentially regulate the three downregulated miRNAs (Supplementary Excel 4, 5). The ceRNA hypothesis assumes that lncRNA can attenuate miRNA activity through chelation, thereby upregulating the expression of miRNA-target genes. Therefore, the eligible lncRNA should negatively correlate with miRNA while positively correlating with target mRNA. Therefore, the predicted expression level and prognostic value of lncRNA were verified using GEPIA and Kaplan–Meier plotter databases, respectively. Only LINC00665 was notably upregulated in LUAD samples when compared to healthy controls (Fig. 6A). Subsequent survival analysis showed that patients with high expression of LINC00665 had poor prognosis (Fig. 6B). By contrast, three lncRNAs (LINC01140, NEAT1, and PCAT19) were significantly downregulated compared to those of healthy controls and displayed a good prognosis (Fig. 6C–F). Therefore, we defined these four as key lncRNAs in the ceRNA network.

### Construction of the lncRNA–miRNA–mRNA regulatory network in LUAD

**Figure 7:**
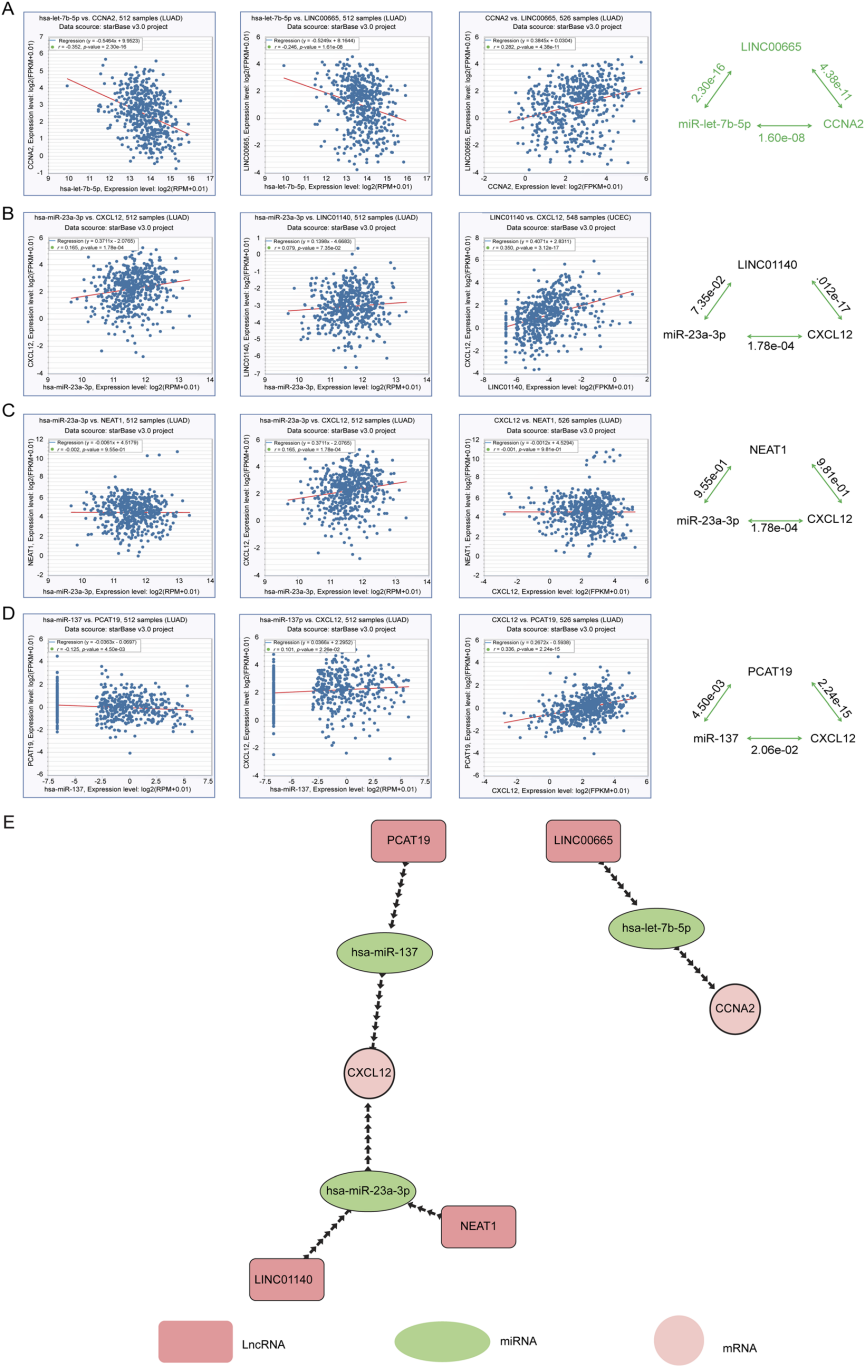
Construction of the lncRNA–miRNA–mRNA regulatory network in LUAD. Correlations within mRNA, miRNA, and lncRNA using the starBase database (**A**–**D**). (**E**) Novel mRNA–miRNA–lncRNA competing endogenous RNA (ceRNA), triple-regulatory network associated with the prognosis of LUAD.

Based on the previous prediction and validation, a vital lncRNA–miRNA–mRNA ceRNA network in patients with LUAD was established. Following the ceRNA hypothesis mentioned above, miRNA expression was inversely correlated with mRNA and lncRNA, whereas lncRNA expression was positively correlated with mRNA. Thus, we further assessed the correlation of interactions between all these RNA pairs in the network by using the starBase database. As shown in Fig. 7A–D, only the LINC00665-miR-let-7b-5p-CCNA2 regulatory network could fit within the ceRNA mechanism. Finally, we constructed a new three sub-networks of mRNA-miRNA-lncRNA, which significantly correlated with the prognosis of LUAD (Fig. 7E). This sub-network can also be used to identify promising diagnostic biomarkers or to develop therapeutic targets for LUAD.

## Discussion

Lung cancer is known for its poor prognosis and high mortality; its metastatic mechanism is complex and remains unclear^24,25^. Poor prognosis for patients with lung cancer prompts us to formulate effective treatment measures and find more effective prognostic indicators^26^. Therefore, revealing molecular switches that control the malignant transformation of lung cancer is of considerable significance, which may also reveal new prognostic indicators. Recent research suggests that ncRNAs, including miRNAs and lncRNAs, play pivotal roles in the occurrence or development of cancer^27,28^. There is increasing evidence of a close, complex relationship between miRNAs and lncRNAs in cancer^29,30^. Based on the ceRNA hypothesis first proposed by Salmena et al.^8^, active research on ceRNAs in human cancer has been implemented, which has shown that they participate in various pathological processes, including tumorigenesis^31,32^. Liu et al. reported that lncRNA-XIST as ceRNA negatively regulates the expression of miR-34a and drives thyroid cancer proliferation and growth via the MET-PI3K-AKT signaling pathway^33^. lncRNA XLOC-006390 also has the function of ceRNA and negatively regulates the expression of miR-331-3p and miR-338-3p, thereby promoting tumorigenesis and metastasis of cervical cancer^34^. Moreover, Gao et al. determined that lncRNA ZEB2-AS1 promotes pancreatic cancer cell growth and invasion by regulating miR-204^35^.

Regarding lung cancer, Schmidt et al. identified the lncRNA, MALAT1, as a prognostic marker for metastasis and patient survival in NSCLC^36^. In addition, Jen and colleagues revealed a new mechanism whereby Oct4 transcriptionally activates lncRNAs, NEAT1 and MALAT1, via promoter and enhancer-binding, respectively, to promote tumor cell proliferation and metastasis, leading to lung tumorigenesis and poor prognosis^37^. Functional validation shows that the largest differentially expressed lncRNA in lung cancer, LCAL1 (lung cancer-associated lncRNA 1), contributes to tumor cell proliferation^38^. Furthermore, siRNA-mediated downregulation of lncRNA and MVIH (microvascular invasion of HCC) can inhibit cell growth by regulating the expression of MMP-2 and MMP-9 proteins in NSCLC^39^.

In our research, we identified two important subsets, including 402 upregulated genes and 638 downregulated genes from three GEO datasets, GSE18842, GSE19188, and GSE33532^40^. GO analysis of these significant DEGs demonstrated that they were significantly enriched in GO terms associated with cancer biological behavior, including cell division^41,42^, cell adhesion^43^, and positive regulation of angiogenesis^44,45^. KEGG pathway enrichment analysis shows that multiple pathways are enriched, mainly p53 signaling pathways and other important regulatory pathways in cancer^46^ and cell cycle-related pathways^47^. More importantly, we found that the deregulation of amino acid metabolism, such as that of pyrimidine, purine, and glutathione, plays vital roles in the reprogramming of cellular metabolism and is essential for tumorigenesis^48,49^. Therefore, these important DEGs may be involved in regulating the occurrence and metastasis of lung cancer.

We hypothesized that these screened DE-mRNAs might interact with each other. Thus, two PPI networks were constructed separately using the STRING database, which displayed complex associations among these DE-mRNAs, especially in the upregulated group. Subsequently, we selected the top 20 upregulated and downregulated hub genes for further expression validation and survival analyses. Convincingly, nearly all hub genes were well-validated in the GEPIA database and proved to be significantly associated with prognosis, implying that they may function as key genes in lung cancer.

Interestingly, some of the hub genes have been widely reported to be involved in cancer. For instance, overexpression of CCNB1 and CDK1 induces tumor growth and metastasis in human breast cancer^50^. CCNA2 modulates CDK6 and MET-mediated cell cycle pathway, and epithelial-mesenchymal transition progression is blocked by miR-381-3p in bladder cancer^51^.

In order to systematically explore potential ceRNAs that regulate the above-mentioned central genes, we first predicted their upstream miRNA based on an experimentally verified miRNA-target interaction database miRTarBase. According to the ceRNA hypothesis and combined with expression and survival analysis, 17 candidate miRNAs were identified as key miRNA, of which two (miR-548b and miR-let-7b) had a good prognosis, and three (miR-17, miR-137 and miR- 23a) had a poor prognosis.

Furthermore, lncRNAs regulate downstream genes by sequestering miRNA, according to the ceRNA mechanism. Therefore, we further predicted the upstream lncRNAs for the key miRNAs. Similarly, after strict expression validation and survival analysis, only four (LINC00665, LINC01140, NEAT1, and PCAT19) out of 624 lncRNAs were screened as key lncRNAs. Interestingly, some interactions found in this network were identified in previous studies. Z, C. et al. determined that LINC00665 expression is significantly upregulated in lung cancer tissues and exerts its oncogenic role by competing with miR-98, and subsequently activating downstream AKR1B10-ERK signaling pathway^52^. Moreover, LINC00665 is important for NSCLC to develop drug resistance, and in cells with acquired gefitinib resistance, downregulation of the LINC00665 gene reverses gefitinib sensitivity in vitro and in vivo^53^.

In conclusion, the ceRNA regulatory network is becoming a research focus in the field of noncoding RNA. Employing stepwise reverse prediction from mRNA to lncRNA, we successfully constructed a potential mRNA-miRNA-lncRNA regulatory network in LUAD. Each component of the ceRNA network is significantly related to the prognosis of patients with lung cancer. More importantly, LINC00665- miR-let-7b- *CCNA2* was identified as a novel key ceRNA network for its possible oncogenic function in lung cancer.

Our findings may provide new insights into the pathogenesis of lung cancer. However, further experimental validation is required. In particular, future work should focus on clarifying the function of LINC00665- miR-let-7b- *CCNA2* axis in LUAD with in vitro and in vivo studies. Additional experiments and large-scale clinical trials are required in the future. We have successfully constructed a silencing plasmid for LINC00665 and plan to investigate the tumor biology effect of the intervention of LINC00665 on lung cancer cells. Parallel silencing of LINC00665 in animal tumor models was performed to investigate whether tumor growth could be inhibited. Further, we clarified the direct regulation effect of LINC00665 on miR-let-7b through luciferase reporting and ChIP experiments. Finally, we used a large number of clinical samples to verify that the LINC00665-miR-let-7b-CCNA2 axis plays an important regulatory role in LUAD patients. We believe that identifying ceRNA networks associated with metastasis or staging of LUAD will be relevant for clinical research and is worthy of further investigation and development.

## Methods

### Datasets collection

We searched for the datasets of lung cancer tissues from the GEO database (http://www.ncbi.nlm.nih.gov/geo/). The clinical characteristics of selected datasets were extracted for further analysis. Finally, three datasets (GSE18842, GSE19188, and GSE33532), were selected for subsequent analyses. To increase the reliability of the differential mRNA screening results, we added the TCGA datasets to the discovery set.

### Differential expression analysis

Three matrix and associated platform annotation files of the aforementioned three GEO datasets were first downloaded from the GEO database. The software package “limma” was used in the R software to identify DEGs from three data sets, taking |log_2_FC|> 1 and an adjusted p-value < 0.05 as the cut-off criterion for differential expression analysis. In addition, VENNY 2.1.0 (http://bioinfogp.cnb) was used to draw the Venn diagram. Common DEGs in the GSE18842, GSE19188, and GSE33532 data sets were redefined as significant DEGs, including significantly up- and downregulated DEGs.

### Functional enrichment analysis

These DE-mRNAs were introduced for GO function annotation and the KEGG pathway enrichment analysis to explore the related functions. The website, DAVID, was used for enriched GO terms and KEGG pathways enrichment analysis. A p-value < 0.05 was considered statistically significant. The R software ggplot2 software package was used to visualize the first 15 enriched GO terms and KEGG pathways.

### Construction of PPI network

Protein–protein interactions were constructed separately for DE-mRNAs using the STRING (Search Tool for Retrieval of Interacting Genes/Proteins) database. PPIs with a combined confidence score > 0.4 were used to construct the PPI network. The images were displayed on a high-resolution monitor during the experiment and were downloaded from the webpage.

### Prediction of miRNA

We used the miRTarbase database to predict upstream miRNAs of key genes, and the database was verified experimentally. We also used the Kaplan–Meier plotter database to further evaluate the prognostic value of these predicted miRNAs.

### Prediction of lncRNA

The upstream potential lncRNAs were predicted using the miRNet database, which provides an integrated tool to comprehensively analyze the interaction between miRNA and its targeted lncRNA. Additionally, we evaluated the prognostic value of these predicted lncRNAs using the Kaplan–Meier plotter database.

### Correlation analysis

Correlation analysis of mRNA/miRNA/lncRNA pairs in lung cancer was performed using the starBase database. Statistical significance was set at p-value < 0.05.

## Supplementary Information

Supplementary Information.

Supplementary Information.

Supplementary Information.

## Data Availability

The data used to support the findings of this study are available from the corresponding author upon reasonable request.

## Abbreviations

ceRNA: Competing endogenous RNA
lncRNAs: Long noncoding RNAs
GEO: Gene Expression Omnibus
PPI: Protein–protein interaction
LUAD: Lung adenocarcinoma
