# Supplementary figures and images for: Identification of LINC00665-miR-let-7b-CCNA2 competing endogenous RNA network associated with prognosis of lung adenocarcinoma
Source: Sci Rep. 2021 Feb 24;11:4434. doi: 10.1038/s41598-020-80662-x (PMC7904782; doi:10.1038/s41598-020-80662-x)

**A** Unregulated DEGs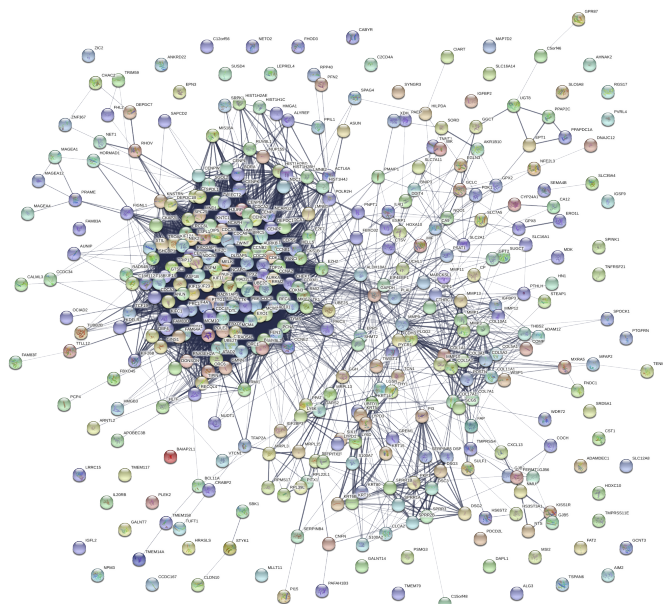**C** Downregulated DEGs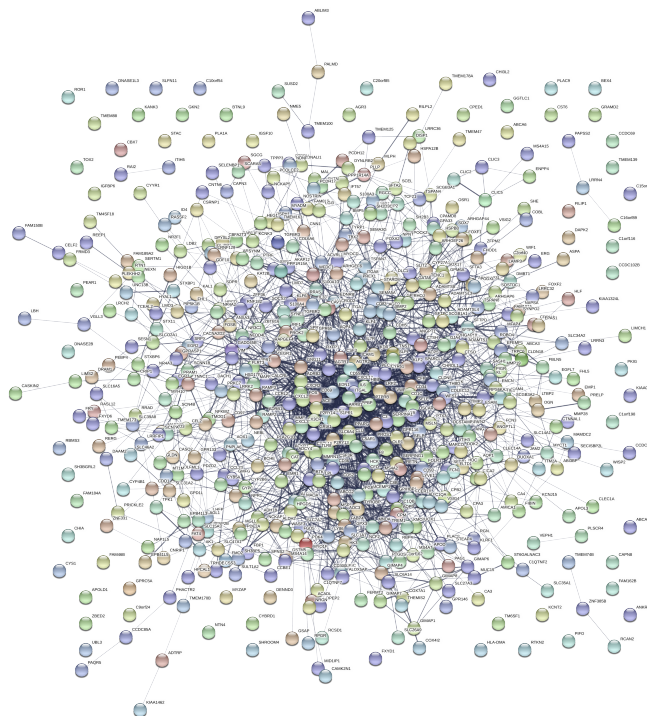**B**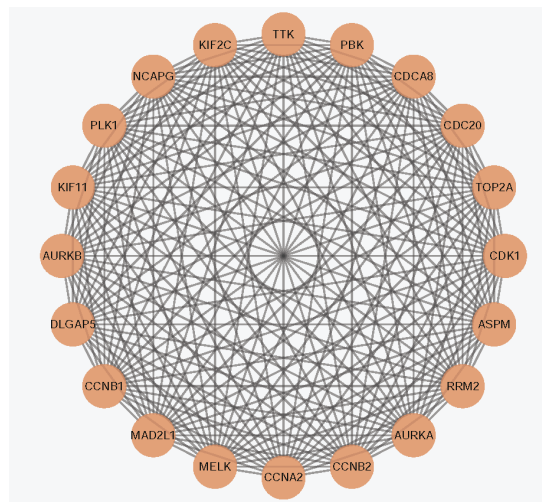**D**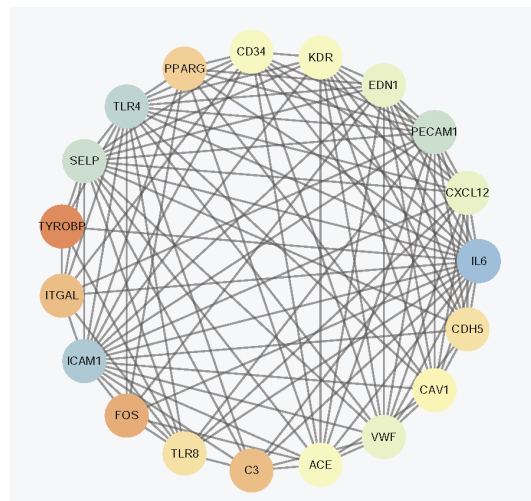

A

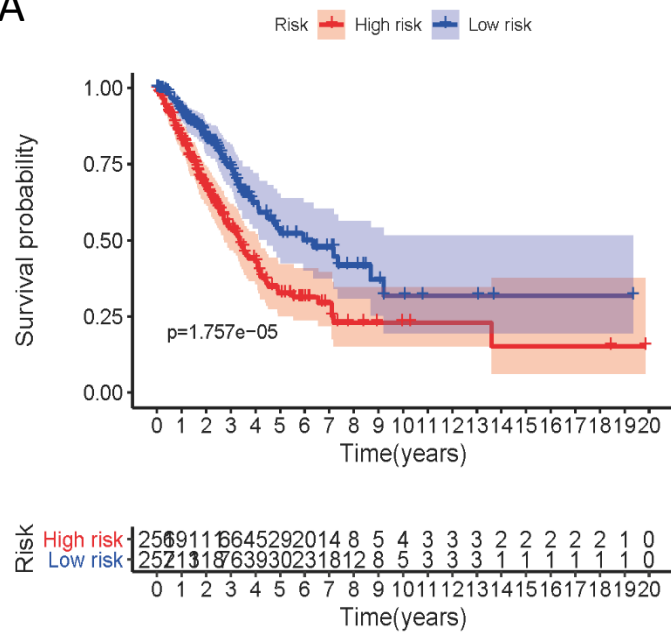

B

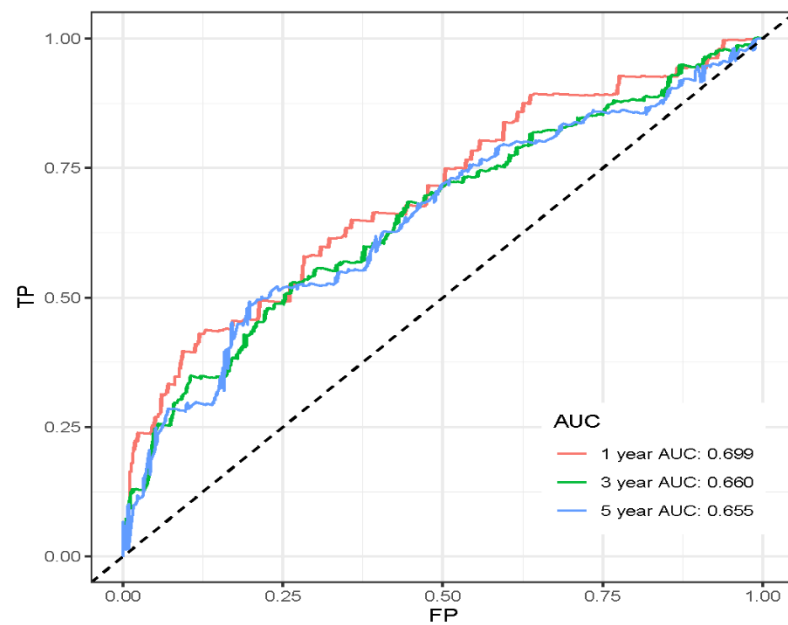

Supplement: Supplementary file 1 — Supplementary Information. [file 41598_2020_80662_MOESM1_ESM.pdf]
