# Supplementary material for: Identification of LINC00665-miR-let-7b-CCNA2 competing endogenous RNA network associated with prognosis of lung adenocarcinoma
Source: Sci Rep. 2021 Feb 24;11:4434. doi: 10.1038/s41598-020-80662-x (PMC7904782; doi:10.1038/s41598-020-80662-x)
Supplement: Supplementary file 3 — Supplementary Information. [file 41598_2020_80662_MOESM3_ESM.docx]

**Supplementary Figure 1. PPI network analysis.**

(A) PPI network of the significantly upregulated DEGs. (B) Top 20 hub genes of the significantly upregulated DEGs. (C) PPI network of the significantly downregulated DEGs. (D) The 20 hub genes of the significantly downregulated DEGs.

**Supplementary Figure 2. Predictive analysis of microRNAs.**

(A) Survival analysis of miRNAs in patients with lung adenocarcinoma. (B) ROC curve of miRNAs in patients with lung adenocarcinoma.

Supplementary Excel 1-GES19188；

Supplementary Excel 2-GSE18842；

Supplementary Excel 3-GSE33532；

Supplementary Excel 4-Lnc-miRNA-downregulated；

Supplementary Excel 5-Lnc-miRNA-upregulated.
